# 4-(4-Nitro­benzene­sulfonamido)pyridinium trichloro­acetate

**DOI:** 10.1107/S160053680800069X

**Published:** 2008-01-16

**Authors:** Peng-Wei Zhang, Wen-Yuan Gao, Li Zhang, Shou-Cheng Pu

**Affiliations:** a School of Pharmaceutical Science & Technology, Tianjin University, Tianjin 300072, People’s Republic of China; b Department of Pharmaceutics, Medical College of Chinese People’s Armed Police Force, Tianjin 300162, People’s Republic of China

## Abstract

In the crystal structure of the title compound, C_11_H_10_N_3_O_4_S·C_2_Cl_3_O_2_, the dihedral angle between the two six-membered rings is 69.2 (1)°. The mol­ecules are connected *via* inter­molecular N—H⋯O hydrogen bonding.

## Related literature

For related literature, see: Talley *et al.* (2000 ▶); El-Naggar *et al.* (1981 ▶).
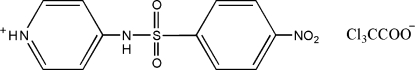


## Experimental

### 

#### Crystal data

C_11_H_10_N_3_O_4_S·C_2_Cl_3_O_2_
*M* *_r_* = 442.65
Monoclinic, 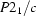

*a* = 22.017 (4) Å
*b* = 6.2187 (12) Å
*c* = 12.719 (3) Å
β = 97.48 (3)°
*V* = 1726.6 (6) Å^3^
*Z* = 4
Mo *K*α radiation
μ = 0.69 mm^−1^
*T* = 113 (2) K
0.14 × 0.12 × 0.04 mm

#### Data collection

Rigaku Saturn diffractometer
Absorption correction: multi-scan (*CrystalClear*; Rigaku/MSC, 2005 ▶) *T*_min_ = 0.910, *T*_max_ = 0.973
9585 measured reflections
3017 independent reflections
2440 reflections with *I* > 2σ(*I*)
*R*_int_ = 0.059

#### Refinement

*R*[*F* ^2^ > 2σ(*F* ^2^)] = 0.051
*wR*(*F* ^2^) = 0.162
*S* = 1.16
3017 reflections
243 parameters
2 restraints
H atoms treated by a mixture of independent and constrained refinement
Δρ_max_ = 0.57 e Å^−3^
Δρ_min_ = −0.54 e Å^−3^

### 

Data collection: *CrystalClear* (Rigaku/MSC, 2005 ▶); cell refinement: *CrystalClear*; data reduction: *CrystalClear*; program(s) used to solve structure: *SHELXS97* (Sheldrick, 2008 ▶); program(s) used to refine structure: *SHELXL97* (Sheldrick, 2008 ▶); molecular graphics: *SHELXTL* (Sheldrick, 2008 ▶); software used to prepare material for publication: *SHELXTL*.

## Supplementary Material

Crystal structure: contains datablocks I, global. DOI: 10.1107/S160053680800069X/nc2087sup1.cif

Structure factors: contains datablocks I. DOI: 10.1107/S160053680800069X/nc2087Isup2.hkl

Additional supplementary materials:  crystallographic information; 3D view; checkCIF report

## Figures and Tables

**Table 1:** Hydrogen-bond geometry (Å, °)

*D*—H⋯*A*	*D*—H	H⋯*A*	*D*⋯*A*	*D*—H⋯*A*
N1—H1⋯O5^i^	0.897 (10)	1.755 (13)	2.639 (4)	168 (4)
N2—H2⋯O6^ii^	0.897 (10)	1.825 (15)	2.707 (4)	167 (4)

Symmetry codes: (i) 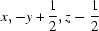; (ii) 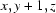.

**Fig. 1.**
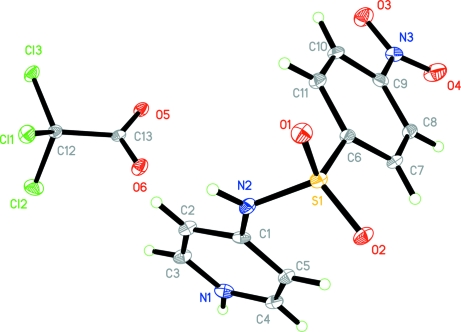
Crystal structure of the title compound with labeling and displacement ellipsoids drawn at the 30% probability level. H atoms are shown as small spheres of arbitrary radii.

## supplementary crystallographic information

### Comment

Benzenesulfonamides are very important intermediates in the organic synthesis and are widely used for the synthesis of pharmaceutical compounds (Talley *et al.*, 2000). In our ongoing investigations on this topic we characterize the the title compound by single-crystal X-ray diffraction. In its crystal structure the dihedral angle between the phenyl and the pyridinyl ring amount to 69.2 (1)°. The 4-nitro-*N*-(pyridinium-4-yl)benzenesulfonamide cations and the trichloroacetate anions are connected by intermolecular N—H···O hydrogen bonding between the N—H atoms of the cations and the oxygen atoms of the anions.

### Experimental

0.5 g(1.8 mmol) of 4-nitro-*N*-(pyridin-4-yl)benzenesulfonamide was dissolved in a mixture of trichloroacetic acid (2.0 mmol,0.33 g) and ethyl acetate (5 ml). Colorless crystals of the title compound were obtained by slow evaporation of the solvent.

### Refinement

The C—H H atoms were positioned with idealized geometry and were refined using a riding model. The N—H H atoms were located in difference map and were refined with varying coordinates and varying isotropic displacement parameters.

### Crystal data

**Table:**

C_11_H_10_N_3_O_4_S_1_·C_2_Cl_3_O_2_	*F*_000_ = 896
*M**_r_* = 442.65	*D*_x_ = 1.703 Mg m^−^^3^
Monoclinic, *P*2_1_/*c*	Mo *K*α radiation λ = 0.71073 Å
*a* = 22.017 (4) Å	Cell parameters from 4622 reflections
*b* = 6.2187 (12) Å	θ = 1.8–28.1º
*c* = 12.719 (3) Å	µ = 0.69 mm^−^^1^
β = 97.48 (3)º	*T* = 113 (2) K
*V* = 1726.6 (6) Å^3^	Lamellar, colorless
*Z* = 4	0.14 × 0.12 × 0.04 mm

### Data collection

**Table:**

Rigaku Saturn diffractometer	3017 independent reflections
Radiation source: rotating anode	2440 reflections with *I* > 2σ(*I*)
Monochromator: confocal	*R*_int_ = 0.059
Detector resolution: 7.31 pixels mm^-1^	θ_max_ = 25.0º
*T* = 111(2) K	θ_min_ = 1.9º
ω scans	*h* = −26→26
Absorption correction: multi-scan(CrystalClear; Rigaku/MSC, 2005)	*k* = −7→7
*T*_min_ = 0.910, *T*_max_ = 0.973	*l* = −15→13
9585 measured reflections	

### Refinement

**Table:**

Refinement on *F*^2^	Secondary atom site location: difference Fourier map
Least-squares matrix: full	Hydrogen site location: inferred from neighbouring sites
*R*[*F*^2^ > 2σ(*F*^2^)] = 0.051	H atoms treated by a mixture of independent and constrained refinement
*wR*(*F*^2^) = 0.162	*w* = 1/[σ^2^(*F*_o_^2^) + (0.0873*P*)^2^] where *P* = (*F*_o_^2^ + 2*F*_c_^2^)/3
*S* = 1.16	(Δ/σ)_max_ = 0.001
3017 reflections	Δρ_max_ = 0.57 e Å^−^^3^
243 parameters	Δρ_min_ = −0.53 e Å^−^^3^
2 restraints	Extinction correction: none
Primary atom site location: structure-invariant direct methods	

### Special details

**Table:**

Geometry. All e.s.d.'s (except the e.s.d. in the dihedral angle between two l.s. planes) are estimated using the full covariance matrix. The cell e.s.d.'s are taken into account individually in the estimation of e.s.d.'s in distances, angles and torsion angles; correlations between e.s.d.'s in cell parameters are only used when they are defined by crystal symmetry. An approximate (isotropic) treatment of cell e.s.d.'s is used for estimating e.s.d.'s involving l.s. planes.
Refinement. Refinement of *F*^2^ against ALL reflections. The weighted *R*-factor *wR* and goodness of fit *S* are based on *F*^2^, conventional *R*-factors *R* are based on *F*, with *F* set to zero for negative *F*^2^. The threshold expression of *F*^2^ > σ(*F*^2^) is used only for calculating *R*-factors(gt) *etc*. and is not relevant to the choice of reflections for refinement. *R*-factors based on *F*^2^ are statistically about twice as large as those based on *F*, and *R*- factors based on ALL data will be even larger.

### Fractional atomic coordinates and isotropic or equivalent isotropic displacement parameters (Å^2^)

**Table:**

	*x*	*y*	*z*	*U*_iso_*/*U*_eq_	
S1	0.18257 (4)	1.36555 (14)	0.07245 (7)	0.0191 (3)	
O1	0.18542 (11)	1.5313 (4)	0.1516 (2)	0.0249 (6)	
O2	0.16464 (11)	1.4166 (4)	−0.03720 (19)	0.0246 (6)	
O3	0.04962 (13)	0.5416 (5)	0.2777 (2)	0.0357 (7)	
O4	−0.00146 (13)	0.5394 (5)	0.1197 (2)	0.0409 (8)	
N1	0.30157 (14)	0.6878 (5)	−0.0407 (3)	0.0231 (7)	
N2	0.25080 (13)	1.2586 (5)	0.0880 (2)	0.0193 (7)	
N3	0.03717 (14)	0.6156 (5)	0.1877 (3)	0.0248 (7)	
C1	0.26602 (16)	1.0661 (5)	0.0425 (3)	0.0182 (8)	
C2	0.31203 (16)	0.9400 (6)	0.0978 (3)	0.0219 (8)	
H2A	0.3310	0.9837	0.1639	0.026*	
C3	0.32893 (16)	0.7525 (6)	0.0544 (3)	0.0241 (8)	
H3	0.3597	0.6688	0.0910	0.029*	
C4	0.25743 (16)	0.8040 (6)	−0.0958 (3)	0.0225 (8)	
H4	0.2395	0.7554	−0.1618	0.027*	
C5	0.23811 (16)	0.9937 (6)	−0.0568 (3)	0.0209 (8)	
H5	0.2071	1.0732	−0.0954	0.025*	
C6	0.13549 (15)	1.1534 (5)	0.1065 (3)	0.0185 (8)	
C7	0.09757 (15)	1.0470 (6)	0.0274 (3)	0.0213 (8)	
H7	0.0944	1.0934	−0.0426	0.026*	
C8	0.06426 (16)	0.8695 (6)	0.0547 (3)	0.0216 (8)	
H8	0.0387	0.7945	0.0034	0.026*	
C9	0.07022 (15)	0.8085 (6)	0.1592 (3)	0.0209 (8)	
C10	0.10690 (16)	0.9146 (6)	0.2399 (3)	0.0219 (8)	
H10	0.1088	0.8706	0.3101	0.026*	
C11	0.14059 (16)	1.0888 (6)	0.2117 (3)	0.0232 (8)	
H11	0.1665	1.1622	0.2632	0.028*	
Cl1	0.43685 (4)	0.58693 (15)	0.39003 (8)	0.0283 (3)	
Cl2	0.46462 (5)	0.17077 (17)	0.30622 (9)	0.0365 (3)	
Cl3	0.41727 (4)	0.20184 (16)	0.50681 (7)	0.0291 (3)	
O5	0.31493 (11)	0.1505 (4)	0.33912 (19)	0.0230 (6)	
O6	0.33887 (11)	0.4175 (4)	0.2360 (2)	0.0265 (6)	
C12	0.41404 (16)	0.3150 (6)	0.3785 (3)	0.0212 (8)	
C13	0.34910 (16)	0.2939 (5)	0.3118 (3)	0.0180 (7)	
H1	0.3107 (17)	0.568 (4)	−0.075 (3)	0.028 (11)*	
H2	0.2756 (17)	1.324 (6)	0.140 (3)	0.047 (13)*	

### Atomic displacement parameters (Å^2^)

**Table:**

	*U*^11^	*U*^22^	*U*^33^	*U*^12^	*U*^13^	*U*^23^
S1	0.0240 (5)	0.0171 (5)	0.0161 (5)	0.0033 (3)	0.0022 (3)	0.0002 (3)
O1	0.0307 (14)	0.0182 (13)	0.0257 (15)	0.0037 (10)	0.0031 (11)	−0.0061 (11)
O2	0.0322 (15)	0.0251 (14)	0.0160 (14)	0.0029 (11)	0.0009 (10)	0.0055 (11)
O3	0.0403 (16)	0.0382 (17)	0.0280 (17)	−0.0053 (13)	0.0023 (12)	0.0112 (14)
O4	0.0468 (18)	0.0483 (19)	0.0276 (17)	−0.0252 (14)	0.0044 (13)	−0.0050 (14)
N1	0.0314 (18)	0.0155 (16)	0.0247 (19)	−0.0008 (13)	0.0120 (13)	−0.0028 (13)
N2	0.0231 (15)	0.0191 (16)	0.0148 (17)	0.0006 (12)	−0.0004 (11)	−0.0027 (13)
N3	0.0277 (18)	0.0256 (17)	0.0220 (19)	0.0001 (13)	0.0067 (13)	−0.0017 (14)
C1	0.0256 (19)	0.0141 (18)	0.017 (2)	−0.0029 (13)	0.0096 (13)	−0.0001 (14)
C2	0.030 (2)	0.0205 (19)	0.015 (2)	0.0007 (14)	0.0023 (14)	0.0000 (15)
C3	0.032 (2)	0.022 (2)	0.019 (2)	0.0021 (15)	0.0053 (15)	0.0035 (16)
C4	0.029 (2)	0.0226 (19)	0.016 (2)	−0.0038 (15)	0.0052 (14)	−0.0004 (15)
C5	0.0251 (19)	0.0208 (19)	0.0166 (19)	0.0009 (14)	0.0016 (14)	0.0026 (15)
C6	0.0191 (18)	0.0209 (19)	0.016 (2)	0.0038 (13)	0.0030 (13)	−0.0014 (14)
C7	0.0237 (19)	0.029 (2)	0.0108 (18)	0.0007 (15)	0.0006 (13)	0.0008 (15)
C8	0.0214 (19)	0.026 (2)	0.016 (2)	0.0010 (14)	0.0002 (14)	−0.0045 (15)
C9	0.0204 (18)	0.0223 (19)	0.021 (2)	0.0028 (14)	0.0071 (14)	−0.0008 (15)
C10	0.026 (2)	0.027 (2)	0.0127 (19)	0.0015 (15)	0.0040 (14)	−0.0007 (15)
C11	0.026 (2)	0.027 (2)	0.017 (2)	0.0007 (15)	0.0013 (14)	−0.0041 (15)
Cl1	0.0313 (5)	0.0241 (5)	0.0291 (6)	−0.0098 (4)	0.0022 (4)	0.0005 (4)
Cl2	0.0349 (6)	0.0404 (6)	0.0364 (7)	0.0111 (4)	0.0135 (4)	−0.0022 (5)
Cl3	0.0325 (5)	0.0345 (6)	0.0187 (5)	−0.0087 (4)	−0.0031 (4)	0.0073 (4)
O5	0.0286 (14)	0.0216 (14)	0.0182 (15)	−0.0052 (10)	0.0010 (10)	0.0034 (10)
O6	0.0330 (15)	0.0242 (14)	0.0209 (15)	−0.0064 (11)	−0.0021 (11)	0.0070 (11)
C12	0.0234 (19)	0.0205 (19)	0.020 (2)	−0.0016 (14)	0.0040 (14)	0.0010 (15)
C13	0.0238 (18)	0.0173 (18)	0.0130 (19)	0.0004 (14)	0.0031 (13)	−0.0033 (14)

### Geometric parameters (Å, °)

**Table:**

S1—O2	1.435 (2)	C4—H4	0.9300
S1—O1	1.436 (3)	C5—H5	0.9300
S1—N2	1.631 (3)	C6—C11	1.388 (5)
S1—C6	1.766 (4)	C6—C7	1.388 (5)
O3—N3	1.231 (4)	C7—C8	1.394 (5)
O4—N3	1.227 (4)	C7—H7	0.9300
N1—C4	1.335 (5)	C8—C9	1.372 (5)
N1—C3	1.341 (5)	C8—H8	0.9300
N1—H1	0.897 (10)	C9—C10	1.387 (5)
N2—C1	1.390 (4)	C10—C11	1.386 (5)
N2—H2	0.897 (10)	C10—H10	0.9300
N3—C9	1.472 (5)	C11—H11	0.9300
C1—C2	1.397 (5)	Cl1—C12	1.765 (4)
C1—C5	1.405 (5)	Cl2—C12	1.777 (4)
C2—C3	1.362 (5)	Cl3—C12	1.770 (4)
C2—H2A	0.9300	O5—C13	1.245 (4)
C3—H3	0.9300	O6—C13	1.231 (4)
C4—C5	1.368 (5)	C12—C13	1.570 (4)
			
O2—S1—O1	120.25 (15)	C1—C5—H5	120.6
O2—S1—N2	109.93 (16)	C11—C6—C7	121.7 (3)
O1—S1—N2	104.59 (14)	C11—C6—S1	118.4 (3)
O2—S1—C6	107.91 (16)	C7—C6—S1	119.7 (3)
O1—S1—C6	109.75 (17)	C6—C7—C8	118.9 (3)
N2—S1—C6	103.08 (16)	C6—C7—H7	120.5
C4—N1—C3	121.4 (3)	C8—C7—H7	120.5
C4—N1—H1	113 (2)	C9—C8—C7	118.2 (3)
C3—N1—H1	126 (2)	C9—C8—H8	120.9
C1—N2—S1	124.7 (2)	C7—C8—H8	120.9
C1—N2—H2	123 (3)	C8—C9—C10	123.9 (3)
S1—N2—H2	112 (3)	C8—C9—N3	118.4 (3)
O4—N3—O3	124.1 (3)	C10—C9—N3	117.7 (3)
O4—N3—C9	117.4 (3)	C11—C10—C9	117.4 (3)
O3—N3—C9	118.4 (3)	C11—C10—H10	121.3
N2—C1—C2	118.2 (3)	C9—C10—H10	121.3
N2—C1—C5	123.4 (3)	C10—C11—C6	119.8 (3)
C2—C1—C5	118.4 (3)	C10—C11—H11	120.1
C3—C2—C1	119.7 (3)	C6—C11—H11	120.1
C3—C2—H2A	120.2	C13—C12—Cl1	110.7 (2)
C1—C2—H2A	120.2	C13—C12—Cl3	112.9 (3)
N1—C3—C2	120.5 (3)	Cl1—C12—Cl3	109.12 (19)
N1—C3—H3	119.7	C13—C12—Cl2	105.4 (2)
C2—C3—H3	119.7	Cl1—C12—Cl2	109.6 (2)
N1—C4—C5	121.1 (3)	Cl3—C12—Cl2	108.90 (19)
N1—C4—H4	119.5	O6—C13—O5	127.6 (3)
C5—C4—H4	119.5	O6—C13—C12	115.6 (3)
C4—C5—C1	118.8 (3)	O5—C13—C12	116.8 (3)
C4—C5—H5	120.6		
			
O2—S1—N2—C1	−62.4 (3)	S1—C6—C7—C8	175.1 (3)
O1—S1—N2—C1	167.2 (3)	C6—C7—C8—C9	0.4 (5)
C6—S1—N2—C1	52.4 (3)	C7—C8—C9—C10	0.8 (6)
S1—N2—C1—C2	−148.4 (3)	C7—C8—C9—N3	−177.4 (3)
S1—N2—C1—C5	32.5 (5)	O4—N3—C9—C8	−11.3 (5)
N2—C1—C2—C3	−178.7 (3)	O3—N3—C9—C8	169.0 (3)
C5—C1—C2—C3	0.4 (5)	O4—N3—C9—C10	170.3 (3)
C4—N1—C3—C2	0.3 (6)	O3—N3—C9—C10	−9.4 (5)
C1—C2—C3—N1	−0.3 (6)	C8—C9—C10—C11	−1.8 (6)
C3—N1—C4—C5	−0.4 (6)	N3—C9—C10—C11	176.4 (3)
N1—C4—C5—C1	0.6 (6)	C9—C10—C11—C6	1.6 (5)
N2—C1—C5—C4	178.5 (3)	C7—C6—C11—C10	−0.4 (5)
C2—C1—C5—C4	−0.6 (5)	S1—C6—C11—C10	−176.2 (3)
O2—S1—C6—C11	−174.3 (3)	Cl1—C12—C13—O6	37.9 (4)
O1—S1—C6—C11	−41.5 (3)	Cl3—C12—C13—O6	160.6 (3)
N2—S1—C6—C11	69.5 (3)	Cl2—C12—C13—O6	−80.6 (3)
O2—S1—C6—C7	9.9 (3)	Cl1—C12—C13—O5	−144.5 (3)
O1—S1—C6—C7	142.6 (3)	Cl3—C12—C13—O5	−21.8 (4)
N2—S1—C6—C7	−106.4 (3)	Cl2—C12—C13—O5	97.0 (3)
C11—C6—C7—C8	−0.6 (5)		

### Hydrogen-bond geometry (Å, °)

**Table:**

*D*—H···*A*	*D*—H	H···*A*	*D*···*A*	*D*—H···*A*
N1—H1···O5^i^	0.897 (10)	1.755 (13)	2.639 (4)	168 (4)
N2—H2···O6^ii^	0.897 (10)	1.825 (15)	2.707 (4)	167 (4)

Symmetry codes: (i) *x*, −*y*+1/2, *z*−1/2; (ii) *x*, *y*+1, *z*.
